# Identification of novel long non-coding RNA biomarkers for prognosis prediction of papillary thyroid cancer

**DOI:** 10.18632/oncotarget.17556

**Published:** 2017-05-02

**Authors:** Qiuying Li, Haihong Li, Lu Zhang, Chunming Zhang, Wentao Yan, Chao Wang

**Affiliations:** ^1^ Department of Otorhinolaryngology, Head and Neck Surgery, The Second Affiliated Hospital of Harbin Medical University, Harbin, China; ^2^ Department of Intensive Care Unit, The Affiliated Hongqi Hospital of Mudanjiang Medical University, Mudanjiang, China; ^3^ Department of Otorhinolaryngology, Hegang People's Hospital, Hegang, China; ^4^ Department of Otorhinolaryngology, The Affiliated Hospital of Northeast Agriculture University, Harbin, China

**Keywords:** biomarkers, long non-coding RNA, papillary thyroid cancer, prognosis

## Abstract

Papillary thyroid carcinoma (PTC) is the most frequent type of malignant thyroid tumor. Several lncRNA signatures have been established for prognosis prediction in some cancers. However, the prognostic value of lncRNAs has not been investigated in PTC yet. In this study, we performed genome-wide analysis of lncRNA expression profiles in a large cohort of PTC patients from The Cancer Genome Atlas and identified 111 differentially expressed lncRNAs between tumor and non-tumor samples and between recurrent and recurrence-free samples. From the 111 differentially expressed lncRNAs, four independent lncRNA biomarkers associated with prognosis were identified and were integrated into a four-lncRNA signature which classified the patients of training dataset into the high-risk group and low-risk group with significantly different overall survival (p=0.016, log-rank test). The prognostic value of the four-lncRNA signature was validated in the independent testing dataset. Multivariate analysis indicated that the four-lncRNA signature was an independent prognostic predictor. Moreover, identified four lncRNA biomarkers demonstrates good performance in predicting disease recurrence with AUC of 0.833 using leave one out cross-validation. Our study not only highlighted the potential role for lncRNAs to improve clinical prognosis prediction in patients with PTC and but also provided alternative biomarkers and therapeutic targets for PTC patients.

## INTRODUCTION

Thyroid cancer is the ninth most common cancer and is the most prevalent cancer among endocrine tumors [1]. The incidence of thyroid cancer has increased rapidly worldwide in the past few decades, representing 1%-2% of the total tumor [2]. Thyroid cancer is composed of three main pathological types of carcinomas: papillary thyroid carcinoma (PTC), follicular thyroid carcinoma (FTC) and anaplastic thyroid carcinoma (ATC) [3]. Papillary thyroid carcinoma (PTC) is the most frequent type of malignant thyroid tumor, comprising 80% of all thyroid cancer cases [3, 4]. Although current treatment by surgery and radioiodine therapy is effective, the number of more advanced tumors and thyroid-cancer-associated mortality is increasing. Therefore, it is critical to identify novel disease biomarkers for improving PTC patient management and making tailored therapeutic decisions.

Recent advancements in the genome and transcriptome analysis revealed that protein-coding genes only comprised ∼2% of the human genome sequences, whereas a substantial fraction of the human genome can be transcribed into non-coding RNAs (ncRNAs) [5]. Long non-coding RNAs, accounting for a large part of the newly discovered ncRNAs, are arbitrarily defined as non-coding transcripts ranging in size from about 200 bp to tens of thousands of bases [6]. Increasing evidence has suggested that lncRNAs are key players of genome regulatory network and play important functional roles in various fundamental biological processes at both the posttranscriptional and transcriptional level [7, 8]. A handful of recent studies has identified have detected a number of dysregulated lncRNAs in cancer tissues or cell lines implying that lncRNAs may possess oncogenic or/and tumor-suppressor properties [9–11]. Like proteins, mRNAs and miRNAs, lncRNAs are emerging as novel molecular biomarkers for early diagnosis and prognosis prediction in various cancers [12–20]. Most recently, thousands of lncRNAs were found to be differentially expressed between PTC and adjacent noncancerous samples [21]. Another study performed by Lan *et al*. also identified thousands of significantly differentially expressed lncRNAs in PTC relative to noncancerous thyroid tissue [22]. These studies made lncRNAs attractive as valuable diagnostic and prognostic biomarkers in PTC. However, the prognostic value of lncRNAs has not been investigated in PTC yet.

The purpose of this study is to identify novel lncRNA biomarkers closely associated with the survival and recurrence of PTC patients from The Cancer Genome Atlas (TCGA), and to assess the prognostic value of novel lncRNA biomarkers for predicting survival and recurrence in patients with PTC.

## RESULTS

### Identification of lncRNA biomarkers associated with prognosis in PTC

**Figure 1:**
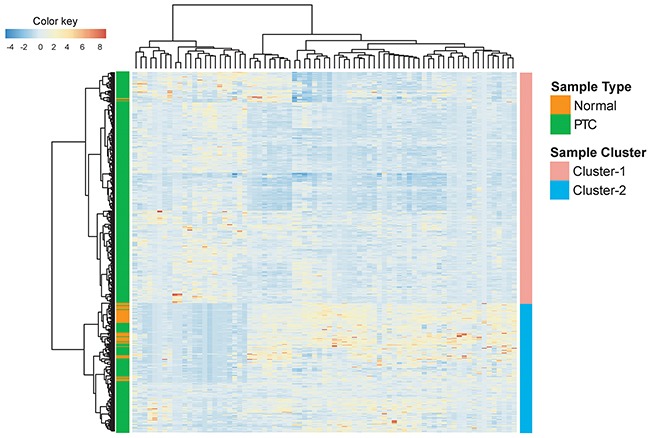
Clustering heatmap visualization of lncRNAs differentially expressed among tumor (n=496) and non-tumor samples (n=59)

We first performed differentially expressed analysis to identify potential lncRNAs associated with prognosis in PTC. Compared with the normal samples, we found that there were 77 differentially expressed lncRNAs (adjusted p-value <0.05). Among them, 22 lncRNAs were up-regulated and 55 lncRNAs were down-regulated in PTC. Hierarchical clustering of 246 PTC patients in the entire TCGA dataset according to the expression patterns of these 77 differentially expressed lncRNAs suggested that these up-regulated and down-regulated lncRNAs could effectively discriminate tumor and non-tumor samples (p<0.001, chi-square test). As showed in Figure 1, Cluster I contained close to the majority of tumor samples (n=355; 71.4%). Conversely, Cluster II contained the majority of normal samples (n=57; 96.6%). Analysis of lncRNA expression profiles in PTC with recurrence compared with those without recurrence identified a total of 34 differentially expressed lncRNAs (adjusted p-value <0.05).

These differentially expressed lncRNAs were subjected to univariate Cox proportional hazard regression analysis to identify lncRNAs, whose expression level could be significantly correlated with patient survival. We identified a set of six lncRNAs (p<0.05) that was significantly correlated with patient survival. We then selected four lncRNAs (*RP11-536N17.1*, *RP11-508M8.1*, *AC026150.8* and *CTD-2139B15.2*) as independent lncRNA biomarkers that were correlated with prognosis by multivariate Cox proportional hazards regression analysis (P<0.05). Table 1 shows a list of these four lncRNAs biomarkers along with important variable information. The positive coefficient of Cox analysis indicated that the higher expression level of four lncRNA biomarkers was associated with poor prognosis.

**Table 1:** LncRNA biomarkers significantly with the prognosis in the training dataset (n=246)

Ensembl ID	Gene symbol	Chromosomal position	Hazard ratio	Z-score	Coefficient	P value
ENSG00000253628.1	RP11-536N17.1	Chr5: 172,816,621-172,819,958(+)	2.903	1.89	1.066	0.048
ENSG00000249365.1	RP11-508M8.1	Chr5: 125,108,204-125,149,929(+)	2.629	2.992	0.967	0.003
ENSG00000260693.1	AC026150.8	Chr15: 30,540,093-30,545,969(+)	3.472	3.414	1.245	0.001
ENSG00000248223.1	CTD-2139B15.2	Chr5: 17,353,910-17,354,899(+)	3.225	3.185	1.171	0.001

### Development and validation of lncRNA signature in survival prediction of PTC in the training dataset

**Figure 2:**
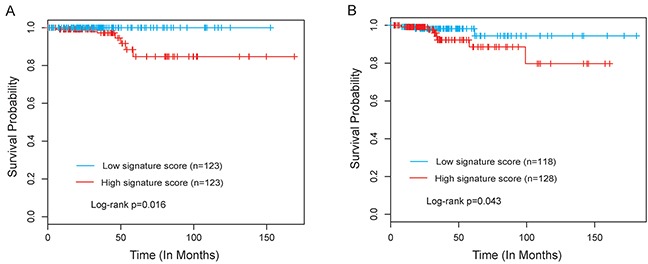
The four-lncRNA signature predicts overall survival of patients with PTC **(A)** Kaplan-Meier survival curves of overall survival between high-risk group and low-risk group in the training dataset. **(B)** Kaplan-Meier survival curves of overall survival between high-risk group and low-risk group in the testing dataset.

We then used these four independent lncRNA biomarkers to construct a lncRNA signature by the risk scoring method as the classifier for survival prediction according to the expression of the four lncRNA biomarkers and using the multivariate Cox regression coefficient as the weight, as follows: lncRNA signature scores= ((1.066 * expression value of *RP11-536N17.1*) + (0.967 * expression value of *RP11-508M8.1*) + (1.245 * expression value of *AC026150.8*) + (1.171 * expression value of *CTD-2139B15.2*)). Using the median lncRNA signature score as risk cutoff value, the lncRNA signature classified 246 PCT patients of training dataset into the high-risk group (n=123) and low-risk group (n=123) with significantly different survival time (p=0.016, log-rank test) (Figure 2A). Furthermore, univariate analysis revealed that the lncRNA signature was significantly associated with overall survival in PTC patients (Table 2). The hazard ratios of high-risk group versus low-risk for overall survival was 1.094 (p=0.004, 95% confidence interval (CI) = 1.028-1.163) (Table 2).

**Table 2:** Univariate Cox regression analyses of the lncRNA biomarkers associated with prognosis in PTCU

Variables		Univariate analysis		
		HR	95% CI of HR	P value
Training dataset (n=261)				
lncRNA signature score	High/low	1.094	1.028-1.163	0.004
Age		1.785	0.323-9.868	0.507
Stage	III, IV/I, II	2.402	0.437-13.207	0.314
Gender	Male/female	2.718	1.625-4.546	P<0.001
Testing dataset (n=246)				
lncRNA signature score	High/low	2.702	1.698-10.451	0.014
Age		1.147	1.082-1.215	P<0.001
Stage	III, IV/I, II	4.469	1.253-15.945	0.021
Gender	Male/female	2.164	0.609-7.689	0.233

### Validation of the lncRNA signature for survival prediction in the testing dataset

To confirm the survival prediction power of the lncRNA signature, we validated the predictive ability of the lncRNA signature in the testing dataset. By using the same risk score formula and risk cutoff value derived from the training dataset, the patients of the testing dataset were classified as high-risk (n=128) or low-risk (n=118) according to the lncRNA signature. Patients in the high-risk group had significantly shorter median survival time than those in the low-risk group (p=0.043, log-rank test) (Figure 2B). In a univariate Cox regression analysis, the hazard ratios of high-risk group versus low-risk for overall survival was 2.702 (p=0.014, 95% CI = 1.698-10.451) (Table 2).

### Survival prediction by the lncRNA signature is independent of clinical factors

To assess whether the prognostic ability of the lncRNA signature in survival prediction is independent of other clinical factors of the patients with PTC, we performed multivariate Cox regression analysis including lncRNA signature, age, stage and gender as covariables. The results from the training dataset showed that the lncRNA signature (HR=1.131, 95% CI=1.01-1.267, p=0.034) and gender (HR=1.151, 95% CI=1.076-1.231, p<0.001) was significantly correlated with overall survival of the patients with PTC (Table 3). In the testing dataset, although univariate analysis revealed that lncRNA signature (HR=2.702, 95% CI=1.698-10.451, p=0.014), age (HR=1.147, 95% CI=1.082-1.215, p<0.001) and stage (HR=4.469, 95% CI=1.253-15.945, p=0.021) were all significantly associated with overall survival, only lncRNA signature (HR=1.151, 95% CI=1.076-1.231, p<0.001) was significant in the multivariate analysis (Table 3). The results of the multivariable Cox regression analysis thus indicated that the prognostic value of the lncRNA signature is independent of other clinical factors for the survival of patients with PTC.

**Table 3:** Multivariate Cox regression analyses of the lncRNA biomarkers associated with prognosis in PTC

Variables		Multivariate analysis		
		HR	95% CI of HR	P value
Training dataset (n=261)				
lncRNA signature score	High/low	1.131	1.01-1.267	0.034
Age		1.722	0.164-18.028	0.65
Stage	III, IV/I, II	0.704	0.064-7.727	0.774
Gender	Male/female	3.424	1.432-8.191	0.006
Testing dataset (n=246)				
lncRNA signature score	High/low	1.151	1.076-1.231	P<0.001
Age		0.673	0.156-2.893	0.594
Stage	III, IV/I, II	0.818	0.1628-4.111	0.807
Gender	Male/female	1.315	0.26-6.652	0.74

### Evaluation of lncRNA biomarkers in predicting the risk of recurrence in PTC

**Figure 3:**
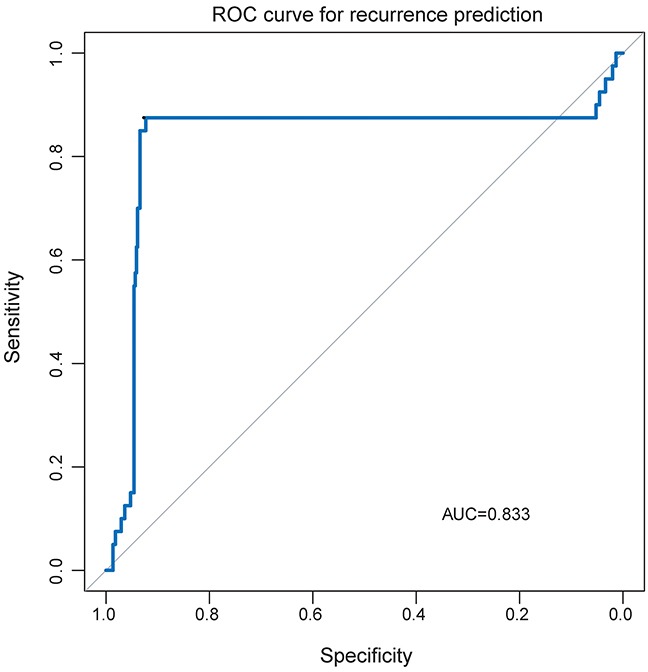
The receiver operating characteristics (ROC) curves of the lncRNA-based classifier in predicting recurrence

We further assessed the capability of the four lncRNA biomarkers in predicting the risk of recurrence in PTC. Thus, we integrated these four lncRNA biomarkers to develop a four-lncRNA classifier using SVM algorithm for measuring how successful the four-lncRNA classifier was in assigning samples to the correct class. The performance of four-lncRNA classifier in predicting recurrence was evaluated using the leave one out cross-validation (LOOCV) procedure. Results of LOOCV procedure showed that the four-lncRNA classifier achieved an overall predictive accuracy of 91.7% with a sensitivity of 87.5% and a specificity of 92.3%. The discriminatory performance of the four-lncRNA classifier, evaluated by calculating the AUC and DOR, revealed that the AUC was 0.833 (Figure 3) and the DOR was 83.909. These results demonstrated that the four-lncRNA classifier had the better predictive performance in predicting recurrence for PTC patients

## DISCUSSION

Papillary carcinoma (PTC) is the most common form of well-differentiated thyroid cancer, and the most common form of thyroid cancer. Although the mean survival rate after 10 years is higher than 90% and most of PTC patients have favorable prognosis especially among patients up to 45 years of age, approximately 5∼20% of patients faced the risk of disease recurrence and suffer aggressive and lethal outcomes. Hence, the use of prognostic biomarkers has become increasingly relevant with the potential goal of improving PTC patient management and making tailored therapeutic decisions. Previous studies establishing the utility of molecular biomarkers in the management of PTC have focused on protein-coding genes or miRNAs in order to identify high-risk patients for more aggressive management. In recent years, increasing evidence have shown that lncRNAs function as a big player in gene regulation and tend to be expressed in a more cell- and tissue-type manner than protein-coding genes, demonstrating the intrinsic advantages of lncRNAs as diagnostic and prognostic biomarkers in cancers [23]. Therefore, development of novel lncRNA biomarkers in prognosis prediction of PTC patients may improve future treatment decisions for PTC patients with regard to the aggressiveness and/or recurrence of the disease.

Several groups have identified thousands of significantly differentially expressed lncRNAs in PTC relative to noncancerous thyroid tissue. In one lncRNA profiling study in three pairs of PTC and adjacent noncancerous samples, 675 lncRNAs were found to be differentially expressed and 8 randomly selected differentially expressed lncRNAs were validated using quantitative polymerase chain reaction (qPCR) [21]. Another study of sixty-two PTC tissue and paired adjacent noncancerous thyroid tissue specimens also identified thousands of significantly differentially expressed lncRNAs [22]. Still, the prognostic signature of lncRNAs in PTC has not been well-established. This study reports the first examination of the prognostic value of lncRNA in a large cohort of more than 100 patients with PTC. The lncRNA expression profiles of noncancerous samples and PTC patients associated with progression and metastasis of patients with PTC were systematically investigated. Differentially expressed lncRNAs between tumor and non-tumor samples and between recurrent samples and recurrence-free samples were identified as previous studies. In order to more effectively construct a specific lncRNA signature for prognosis prediction for PTC patients, we performed univariate and multivariate Cox proportional hazards regression analysis and identified four independent lncRNA biomarkers from most significantly altered lncRNAs. These four independent lncRNA biomarkers can potentially be used to identify patients who are at high-risk of developing PTC recurrence. Then a linear combination of four independent lncRNA biomarkers (*RP11-536N17.1*, *RP11-508M8.1*, *AC026150.8* and *CTD-2139B15.2*) was constructed to form lncRNA signature which was closely related to survival of patients with PTC. The prognostic value of this signature was verified in the training dataset of 246 PTC patients and in an independent testing dataset of 246 PTC patients. Furthermore, the prognostic value of the four-lncRNA signature was independent of clinical factors by performing multivariable Cox regression analysis. We further evaluated the clinical relevance of the four independent lncRNA biomarkers as a predictor in the risk evaluation of recurrence using SVM algorithm. Our findings demonstrated the feasibility and potential power of the four independent lncRNA biomarkers in predicting the risk of recurrence.

Some limitations should be acknowledged for this study. First, roles of four lncRNA biomarkers in PTC pathogenesis are presently unclear. Additional experimental investigations of these four lncRNAs should be conducted to improve our understanding of the biological behavior of three four lncRNAs in PTC. Second, PTC patients used in this study were obtained from a single source (TCGA) and randomly assigned to training and testing datasets for the discovery and validation. So, prognostic lncRNA biomarkers identified here should be validated in independent external PTC datasets. Despite these drawbacks, our study highlighted the potential role for lncRNAs to improve clinical prognosis prediction in patients with PTC. As pointed and discussed in some recent papers [24], the web server of the proposed method is very important. In this regard, we will focus on establishing a web site of the proposed methods in our future studies.

In conclusion, the present study performed genome-wide analysis for lncRNA expression in a large cohort of PTC patients from TCGA and showed altered expression patterns between tumor and non-tumor samples and between recurrent samples and recurrence-free samples. A lncRNA signature comprising four lncRNAs (*RP11-536N17.1*, *RP11-508M8.1*, *AC026150.8* and *CTD-2139B15.2*) were identified, which can be used an independent prognostic marker to robustly predict survival and recurrence of patients with PTC. With further independent validation studies in prospective cohorts, these identified lncRNAs might serve as alternative biomarkers and therapeutic targets for PTC patients.

## MATERIALS AND METHODS

### Patients and clinicopathological data

Clinical and pathological data of 496 patients with PTC were retrieved from The Cancer Genome Atlas (TCGA) (https://cancergenome.nih.gov/). All PTC patients were randomly divided into two distinct sets of equal size: the 246-patient training dataset for discovery purpose and the 246-patient testing dataset for validation purpose. There was no significant difference in clinical and pathological characteristics between patients in the training dataset and those in the testing dataset. Detailed clinical and pathological of patients with PTC used in this study was shown in Table 4.

**Table 4:** Clinicopathological characteristics of the PTC patients used in this study

Covariates		Entire TCGAdataset (n=492)	Trainingdataset (n=246)	Testing dataset(n=246)	P value
Age	<45	225 (45.7%)	121 (49.2%)	104 (42.3%)	0.148^a^
	>=45	267 (54.3%)	125 (50.8%)	142 (57.7%)	
Nodal involvement	With nodal involvement	379 (77%)	197 (80.1%)	182 (74%)	
	Without nodal involvement	4 (0.8%)	1 (0.4%)	3 (1.2%)	0.208
	Unknown	109 (22.2%)	48 (19.5%)	61 (24.8%)	
Extrathyroid extension	None	328 (66.7%)	163 (66.3%)	165 (67.1%)	^b^
	Minimal (T3)	130 (26.4%)	67 (27.2%)	63 (25.6%)	
	Moderate/advanced (T4a)	17 (3.5%)	8 (3.3%)	9 (3.7%)	0.837
	Very advanced (T4b)	1 (0.2%)	1 (0.4%)	0 (0%)	
	Unknown	16 (3.3%)	7 (2.8%)	9 (3.7%)	
Gender	Male	132 (26.8%)	69 (28%)	63 (25.6%)	0.611^b^
	Female	360 (73.2%)	177 (72%)	183 (74.4%)	
Disease free status	With tumor	40 (8.1%)	20 (8.1%)	20 (8.1%)	^b^
	Tumor-free	442 (89.8%)	223 (90.7%)	219 (89%)	0.441
	Unknown	10 (2%)	3 (1.2%)	7 (2.8%)	
Pathologic stage	Stage I	331 (67.3%)	172 (69.9%)	159 (64.6%)	^b^
	Stage II	50 (10.2%)	20 (8.1%)	30 (12.2%)	
	Stage III	107 (21.7%)	51 (20.7%)	56 (22.8)	0.315
	Stage IV	2 (0.4%)	2 (0.8%)	0 (0)	
	Unknown	2 (0.4%)	1 (0.4%)	1 (0.4%)	
Histological type	Other, specify	7 (1.4%)	3 (1.2%)	4 (1.6%)	0.421^b^
	Classical/usual	349 (70.9%)	177 (72%)	172 (69.9%)	
	Follicular	101 (20.5%)	45 (18.3%)	56 (22.8%)	
	Tall cell	35 (7.1%)	21 (8.5%)	14 (5.7%)	
Vital status	Alive	476 (96.7%)	240 (97.6%)	236 (95.9%)	0.446^b^
	Dead	16 (3.2%)	6 (2.4%)	10 (4.1)	

^a^Student's t-test

^b^Chi square test

### Genome-wide lncRNA expression data in normal and tumor samples

Genome-wide lncRNA expression data of 496 PTC samples and 59 normal samples with PTC were obtained from the TANRIC database (http://ibl.mdanderson.org/tanric/_design/basic/download.html) [25]. Briefly, those lncRNAs from the GENCODE Resource (version 19) that overlapped with any known coding genes were filtered out which resulted in 12727 lncRNAs. Then expression levels of lncRNAs were quantized as reads per kilobase per million mapped reads (RPKM) values using TCGA RNA-sequencing data in the BAM file.

### Differentially expressed analysis

A paired student t-test was used to identify differentially expressed lncRNAs between normal samples and PTC samples or between samples with recurrence and samples without recurrence. LncRNAs with an adjusted P-value <0.05 after Benjamini and Hochberg correction were considered as differentially expressed lncRNAs. Hierarchical clustering of the expression values of differentially expressed lncRNAs was performed with R software using the metric of euclidean distance and complete linkage.

### Development of lncRNA signature in survival prediction

Univariate Cox analysis was used to investigate the relationship between the continuous expression level of each lncRNAs and survival. Those lncRNAs with p-value <0.05 were selected as candidate lncRNA biomarkers. Then those candidate lncRNA biomarkers were fitted in the multivariate Cox analysis to identify independent lncRNA biomarkers. Finally, a lncRNA signature was constructed by the linear combination of the expression levels of independent lncRNA biomarkers with the multivariate Cox regression coefficient as the weight. This lncRNA signature could calculate an expression-based risk score for each patient and classify patients into high-risk group and low-risk group using the median risk score from the training dataset

### Survival analysis

Kaplan-Meier survival curves and log-rank tests were used to assess the differences in survival time between the high-risk and low-risk patients. Univariate and multivariate analyses with Cox proportional hazards regression for survival were performed on the individual clinical variables with and without the lncRNA signature in the training dataset and testing dataset. Hazard ratios (HR) and 95% confidence intervals (CI) were calculated.

### Development of lncRNA-based classifier in recurrence prediction

For classification of samples with recurrence or samples without recurrence, independent lncRNA biomarkers were integrated to form classifier using support vector machine (SVM) with the sigmoid kernel [26]. An unbiased performance estimate of the lncRNA-based classifier was carried out using leave one out cross-validation (LOOCV). Diagnostic ability of the lncRNA-based classifier was evaluated by obtaining the area under a receiver operating characteristic (ROC) curve (AUC) and diagnostic odds ratio (DOR). The ROC curve was produced by plotting true positive rates (sensitivity) against false positive rates (1-specificity). The DORs were calculated as follows:

DOR=(Sensitivity×Specificity)/((1−Sensitivity)×(1−Specificity))
